# *Lactobacillus murinus* HF12 colonizes neonatal gut and protects rats from necrotizing enterocolitis

**DOI:** 10.1371/journal.pone.0196710

**Published:** 2018-06-22

**Authors:** Mubina Isani, Brandon A. Bell, Patrick T. Delaplain, Jordan D. Bowling, Jamie M. Golden, Melissa Elizee, Laura Illingworth, Jin Wang, Christopher P. Gayer, Anatoly V. Grishin, Henri R. Ford

**Affiliations:** 1 Division of Pediatric Surgery, Children’s Hospital Los Angeles, Los Angeles, California, United States of Americafs; 2 Department of Surgery, Keck School of Medicine, University of Southern California, Los Angeles, California, United States of America; Gaziosmanpasa University, TURKEY

## Abstract

The use of lactobacilli in prevention of necrotizing enterocolitis (NEC) is hampered by insufficient knowledge about optimal species/strains and effects on intestinal bacterial populations. We therefore sought to identify lactobacilli naturally occurring in postnatal rats and examine their ability to colonize the neonatal intestine and protect from NEC. *L*. *murinus*, *L*. *acidophilus*, and *L*. *johnsonii* were found in 42, 20, and 1 out of 51 4-day old rats, respectively. Higher proportion of *L*. *murinus* in microbiota correlated with lower NEC scores. Inoculation with each of the three species during first feeding significantly augmented intestinal populations of lactobacilli four days later, indicating successful colonization. *L*. *murinus*, but not *L*. *acidophilus* or *L*. *johnsonii*, significantly protected against NEC. Thus, lactobacilli protect rats from NEC in a species- or strain-specific manner. Our results may help rationalizing probiotic therapy in NEC.

## Introduction

Necrotizing enterocolitis (NEC), a severe intestinal inflammation affecting the pre-term infants, remains a leading cause of neonatal morbidity and mortality [1, 2]. It affects 0.5–5 out of every 1000 live births and accounts for 7.7% of neonatal intensive care unit admissions [3, 4]. Although the exact etiology of NEC is unknown, putative risk factors include prematurity, perinatal insults such as hypoxia, hypothermia, and enteral formula feeding, as well as bacterial colonization of the gut [5, 6]. Long-term complications of NEC include intestinal strictures, short bowel syndrome, neurodevelopmental delay, and growth retardation [7].

Although it is generally agreed that the intestinal microbiota plays a key role in the pathogenesis of NEC, the nature of the relationship between NEC and specific groups of bacteria and characteristics of bacterial populations remains unclear. Early bacterial populations of neonates depend on environmental factors such as mode of delivery, formula feeding vs. breast feeding, antibiotic exposure, and others [8]. The immature intestinal epithelium and immune system in pre-term neonates lead to so-called “leaky gut”, a condition that allows luminal bacteria to translocate across the epithelium and trigger an inflammatory cascade that may culminate in NEC [5, 9, 10].

The fact that NEC prevention is preferable to treatment has stimulated a search for possible prophylactic interventions. One such intervention, administration of probiotics, has been extensively investigated [11–13]. Probiotics are live microorganisms whose administration has beneficial effects [14]. Meta-analysis of clinical data demonstrated that probiotics may reduce the severity and mortality from NEC [15]. However, probiotic administration protocols were not standardized across trials. Furthermore, the choice of specific probiotic species was rather arbitrary, and dosages of bacteria varied greatly [9, 15, 16]. Empirical probiotic therapy may carry its own risks. In fact, a few case reports have implicated certain species of bacteria given as probiotics, such as *Lactobacillus rhamnosus*, as a cause of sepsis in neonates [17].

In an effort to rationalize probiotic use in NEC, we have been identifying bacteria in the rat model of NEC and characterizing their ability to colonize the neonatal intestine and influence the disease. *Cronobacter muytjensii* 51329 [18] and *E*. *coil* EC25 [19] are examples of bacteria that exacerbate NEC or protect from NEC, respectively. In this report, we focus on naturally occurring lactobacilli. We have found a strain of *L*. *murinus*, which acts as an effective first colonizer and protects against experimental NEC.

## Materials and methods

### NEC model

The Institutional Animal Care and Use Committee (IACUC) and Institutional Biosafety Committee (IBC) at Children’s Hospital Los Angeles specifically approved this study. Neonatal rats were obtained from timed-pregnant Sprague Dawley dams purchased from Envigo (Placentia, CA) or Charles River Laboratories (Hollister, CA). The newborn rats were separated from their mothers immediately after birth and kept in a temperature (30°C) and humidity (90%) controlled baby incubator (Ohio Medical Products, Madison, WI). NEC was induced according to our previously published protocol [3, 5, 20, 21]. Shortly, the neonates were fed by oral gavage with 200 μl of formula (15 g Similac 60/40, Ross Pediatrics Columbus, OH) in 75 ml of Esbilac canine milk replacement, Pet-Ag Inc., Hampshire, IL) 4 times daily for 4 days. Measures were taken not to introduce extraneous bacteria during handling and feeding (gowning, face mask, gloves, sterile catheters). Fresh formula was prepared daily; each new batch was tested for bacterial contamination by plating on blood agar and MRS. Hypoxia (10 min at 5% O_2_ and 95% N_2_) was administered after each feeding. Bacteria were added to formula from fresh overnight cultures. *Cronobacter muytjensii* 51329 was purchased from ATCC (Manassas, VA). On day 4, terminal ileum samples were removed, fixed in formalin, embedded in paraffin, and sectioned. The sections were stained with hematoxylin-eosine and scored for NEC by a pathologist blinded to treatment groups on a 5-point scale where 0 is no pathology; 1, epithelial sloughing and/or mild sub-mucosal edema; 2, damage to the tips of the villi and/or extensive sub-mucosal edema; 3, damage extending beyond half-way of the length of the villi; and 4, complete obliteration of the epithelium (S1 Fig). Rat pups were euthanized by decapitation following pentobarbital anesthesia, and adult animals by CO_2_ asphyxia.

### Analysis of bacterial populations

To identify and enumerate intestinal bacteria, the content of the freshly excised small intestine was serially diluted on ice and plated on blood agar (Sigma, St. Louis, MO) and MRS agar (Oxoid, Basinstoke, UK). Sample freezing was avoided, as it reduced bacterial viability. After 4-day incubation at 37°C under aerobic condition (blood agar) or in the atmosphere of CO_2_ (MRS agar) the emerging colonies were classified according to their appearance, and numbers in each class were counted. Pure cultures were established for each colony class by re-streaking and maintained as frozen stocks. To identify bacteria, 16S ribosomal RNA gene fragment sequence was PCR-amplified using the 27F and 1492R primers, PCR products sequenced at GeneWiz (Los Angeles, CA), and sequences queried against NCBI non-redundant nucleotide database (nt) using the BLAST algorithm. Gram staining was performed according to the standard protocol. To establish presence of strict anaerobes, stab inoculation into the thioglycollate medium (Becton Dickinson, Franklin Lakes, NJ) was used.

For initial set of samples, bacterial populations were also characterized by high throughput 16S sequencing of DNA from intestinal content, and bacterial loads were determined by real time PCR. For these purposes, DNA was extracted from the same samples that were used for culture analysis by vortexing with 200 micron glass beads in TEN buffer (10 mM Tris pH 8.0, 100 mM NaCl, 1 mM EDTA), adding sodium dodecyl sulfate and Proteinase K to 1% and 20 μg/ml respectively, and incubating for 5 h at 50°C. DNA was further purified by deproteinization with equilibrated phenol pH 8.0, extraction with chloroform, and precipitation with ethanol. 16S RNA segment in each DNA fragment was amplified with one of the pairs of primers with 7 bp barcodes. The resulting amplification products were mixed and random PCR products were sequenced and analyzed at the Children’s Hospital Los Angeles genome core facility using the Illumina MiSeq platform and sequences were analyzed using QIIME software. 16S sequences that passed FastQC and chimera quality controls were clustered into operational taxonomic units (OTUs) corresponding to bacterial genera. Sequences were assigned to samples by barcodes. The output was percentages of different bacterial genera in each sample. Quantitative PCR for determining bacterial loads was performed using 16S primers 27F and 534R and SYBR Green Master Mix (Bio-Rad, Hercules, CA) on Light Cycler 480 (Roche Molecular Diagnostics, Pleasanton, CA) using the following cycling conditions: initial denaturing at 95°C for 1 min, then repeated 95°C 5 sec, 55°C 5 sec, and 70°C 30 sec. PCR specificity was verified by recording product melting curves. Bacterial concentrations were deduced by interpolation using calibrating curves obtained with samples containing known numbers of *E*. *coli* cells.

### Bacterial culture

Lactobacilli were grown in MRS broth at 37°C and 200 rpm on orbital shaker. Care was taken to minimize exposure to air. Upon inoculation, containers were flushed with CO_2_ and tightly sealed. *C*. *muytjensii* was grown aerobically in Luria-Bretani broth at 37°C. Culture OD_600_ was measured using spectrophotometry; correspondence between OD_600_ and cfu/ml was established by serial dilution and plating. In animal inoculation experiments, control platings were done to ascertain the actual dose of live bacteria.

### Restriction digests of bacterial DNA

Bacterial DNA was isolated as described above. 5 μg DNA samples were digested with 10 u *Hin*dIII (New England Biolabs, Ipswich, MA) for 2 h at 37°C, as recommended by the manufacturer. Digestion products were resolved by electrophoresis through 0.8% agarose Tris-acetate gel. Upon staining with ethidium bromide, images were acquired using GelDoc XR (Bio-Rad).

### Immunofluorescence microscopy

Intestinal samples were fixed in buffered formalin, dehydrated, and embedded in paraffin according to the standard procedure. 4 μm sections were mounted on slides, deparaffinized, and incubated in 10 mM Na-citrate buffer pH 7.0 at 110°C for 30 min. Samples were blocked with normal donkey serum in Tris-buffered saline– 0.1% Tween-20, incubated with 1:50 dilution of primary antibodies (Cox-2, Cayman Chemical, Ann Arbor, MI; Mpo, Santa Cruz Biotechnology, Santa Cruz, CA), washed with blocking solution, and incubated with 1:200 dilution of secondary FITC-conjugated donkey anti-rabbit or donkey anti-mouse antibodies (Jackson ImmunoResearch, West Grove, PA). Slides were mounted in Vectashield medium with DAPI (Vector Laboratories, Burlingame, CA). Images were acquired using BX-51 microscope with CCD camera and Picture Frame software (Olympus USA, Center Valley, PA). For comparisons, images were taken at the same exposure, and adjustments applied, if any, were identical. To distinguish between immunofluorescence and background autofluorescence, control samples were processed with omission of the primary antibody. Active caspase-1 was detected on cryosections using the FAM-FLICA kit (Sigma, St. Louis, MO), as recommended by the manufacturer. With considerable autofluorescence, specific signal was revealed by merging images in specific (green) and non-specific (red) channel, in which case specific signal appeared as emerald green, and non-specific as hues of yellow-orange-brown.

### Quantitative RT-PCR

Total RNA was isolated from ileal mucosa scrapings using Trizol (Thermo Fisher Scientific, Waltham, MA). First strand cDNA was synthesized using the iScript kit (Bio-Rad). Quantitative PCR for COX-2 mRNA was performed as described for bacterial 16S RNA gene, using primers ATGTGCACTACGGTTACAAAAGT and TGAACTCTCTCCTCAGAAGAACC. mRNA levels were normalized to those in animals treated with 10^6^ cfu *C*. *muytjensii*.

### Statistical analysis

Data outliers were eliminated by the robust regression and outlier removal (ROUT) method with a Q value of 0.5%. Parametric and non-parametric data were compared by Student’s unpaired *t*-test and χ^2^ test, respectively, using GraphPad Prism software. A p-value of 0.05 or lower was considered significant.

## Results

### Lactobacilli are naturally occurring first colonizers of the intestine in neonatal rats

Since lactobacilli are largely considered beneficial for the health of the GI tract, we sought to examine their prevalence and identity of representative strains of this genus that act as first colonizers of the neonatal rat intestine. Intestinal contents from 4-day-old rats subjected to the NEC-inducing regimen of formula feeding and hypoxia were plated on blood agar to isolate a broad variety of bacteria, as well as on MRS agar, a medium optimized for lactic bacteria. The colonies emerging after 4-day incubation under aerobic (blood agar) or microaerobic (MRS agar) conditions were classified according to their morphology, and representatives of each class were further examined by Gram staining and 16S rRNA sequencing. The lactobacilli grew poorly on blood agar, forming very small colonies with no viable cells. They grew well on MRS agar, forming round white opaque or semi-transparent colonies. On microscopic examination, they appeared as Gram-positive or mixed, non- spore-forming rods of various length, sometimes forming chains. Other bacteria growing on MRS agar were cocci; those were different from the lactobacilli in both colony appearance and cell shape.

In addition to culture-based characterization, bacterial populations in the same intestinal samples were characterized using high throughput sequencing of a variable region of 16S rRNA gene. Both methods yielded similar results with regard to bacteria identified (S1 Data File), but there were several discrepancies in relative proportions of different groups, which could be due to known biases of both culture-dependent and culture-independent methods [22, 23]. Importantly, the culture method yielded bacterial cultures for further characterization, which was the objective of this study.

Microbiome analysis revealed considerable diversity of bacterial populations with regard to species composition, predominant colonizers, and overall loads. This diversity was observed not only among animals from different litters, but among littermates as well, indicating loose relationship between maternal and early postnatal microbiota.

**Fig 1:**
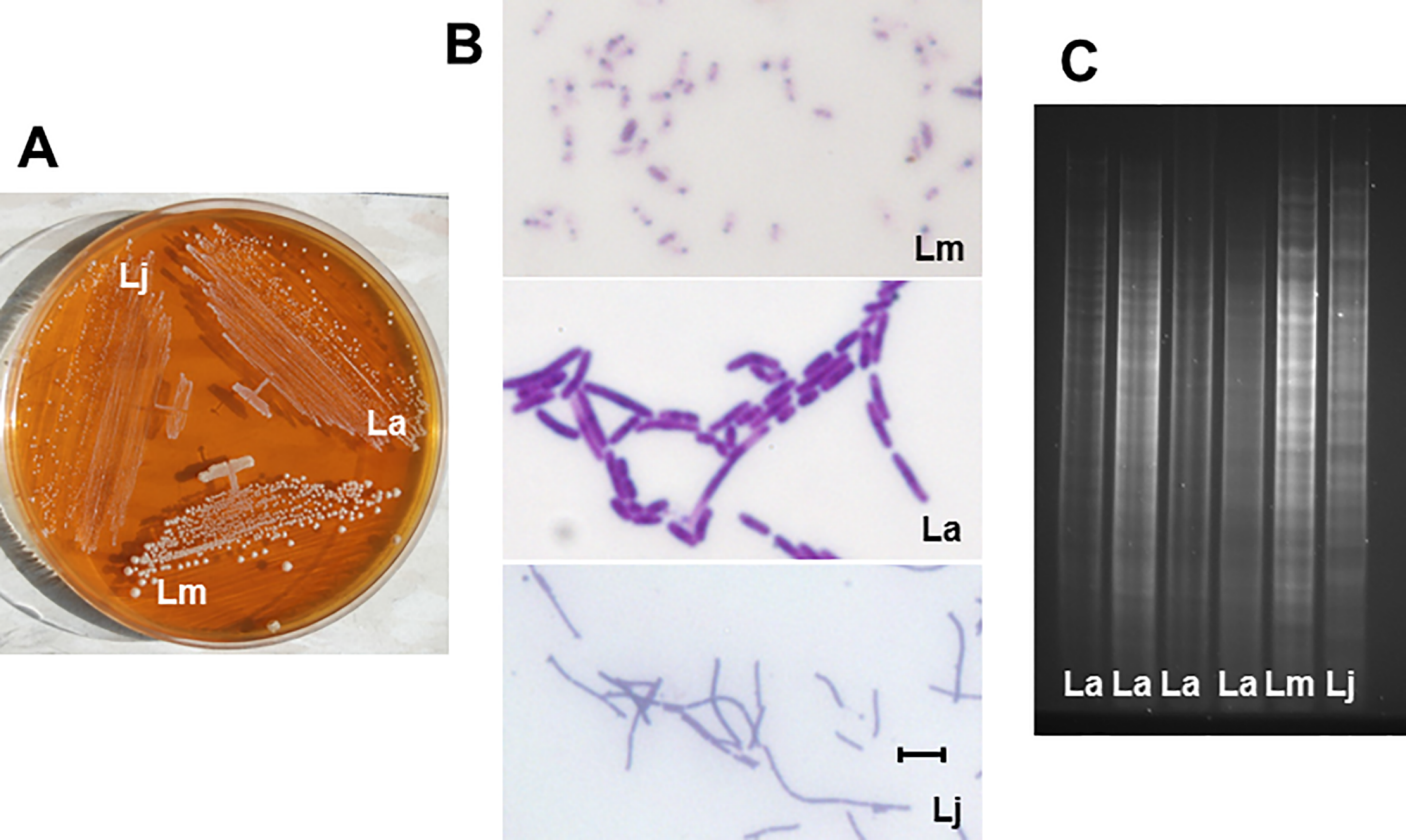
Distinguishing features of *Lactobacillus* strains. A, Colony appearance on MRS agar following 3 day incubation. Note opaque, glassy, and intermediate colonies of *L*. *murinus*, *L*. *johnsonii*, and *L*. *acidophilus*, respectively, as well as differences in colony size. B, microscopic images of Gram-stained bacteria. *L*. *murinus*, *L*. *acidophilus*, *and L*. *johnsonii* are short, stubby intermediate, and thin long noodle-like rods, respectively. C, Electropherograms of *Hin*dIII-digested DNA. Lm, *L*. *murinus*; La, *L*. *acidophilus*; Lj, *L*. *johnsonii*. Bar = 2 μM.

Lactobacilli were present in the vast majority of the animals (47 out of 51). Of the three strains identified, *L*. *murinus* (or possibly *L*. *animalis*) HF12 was the most common (42 animals), *L*. *acidophilus* HF20 was less common (24 animals), and *L*. *johnsonii* HF57 was found in only one animal (S1 Data File). Importantly, distinct colony morphology and microscopic appearance of the three identified *Lactobacillus* strains allowed distinguishing them from other bacteria (Fig 1A and 1B). The three identified strains of lactobacilli had distinct patterns of *Hin*dIII DNA fragments (Fig 1C). All examined isolates of the same species had identical patterns of *Hin*dIII DNA fragments, and were therefore presumed to be the same strain.

Lactobacilli were the predominant bacteria (>50%) in 17 animals. Lactobacilli dominated the microbiomes of the four control breast-fed animals (S1 Data File), although the significance of this finding remains unclear. Thus, in our study group, lactobacilli were common first colonizers, and *L*. *murinus* HF12 was the most frequently found *Lactobacillus* strain.

### Prevalence of *L*. *murinus* HF12 is associated with low NEC scores

**Fig 2:**
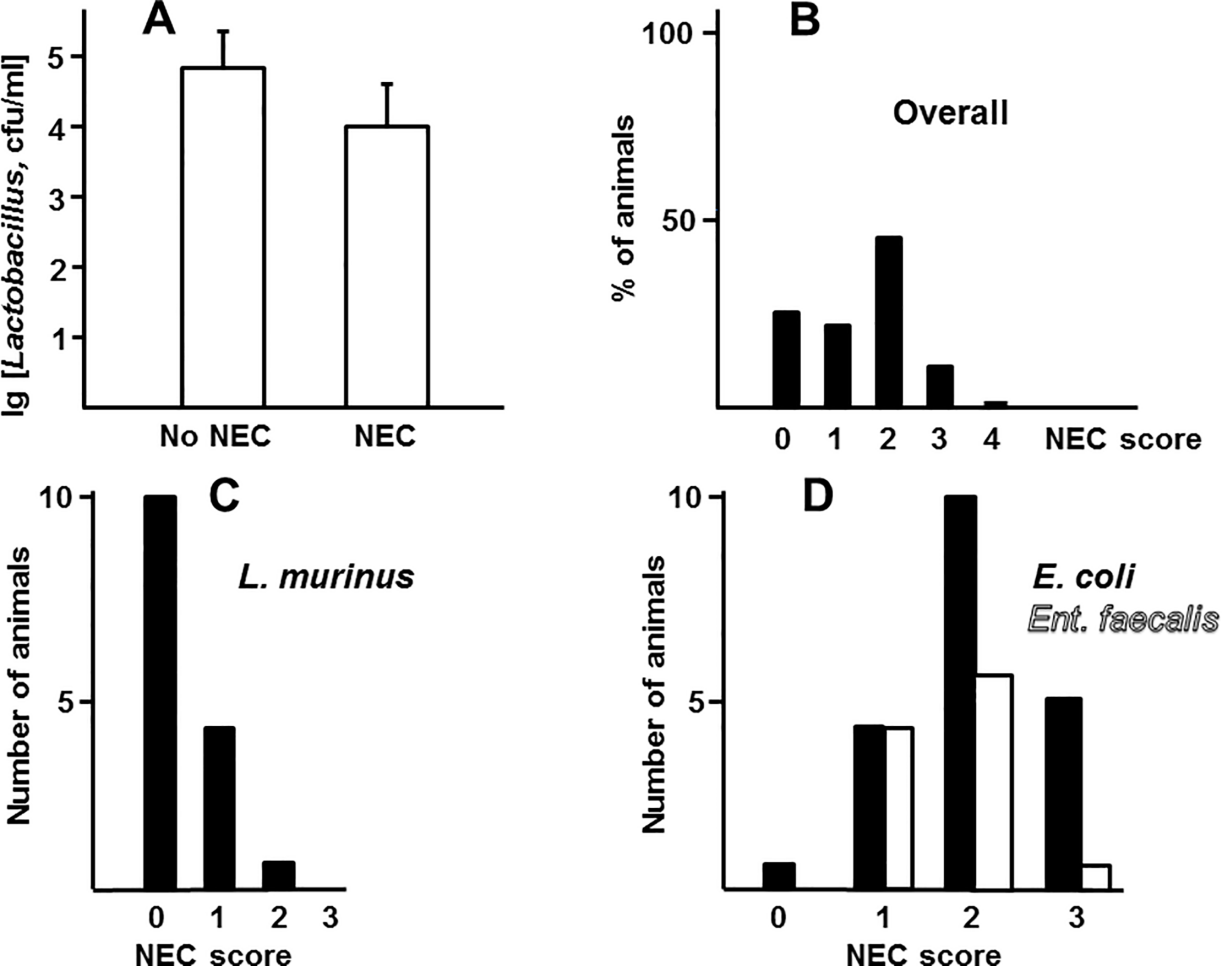
Association of NEC with lactobacilli. A, average logarithms of *Lactobacillus* loads in healthy (NEC scores 0–1, n = 18) and sick (NEC scores 2–4, n = 28) animals. Differences between groups are not significant, p = 0.36, unpaired Student’s *t* test. B-D, distribution of NEC scores in the whole study cohort (B, n = 46) and in groups harboring *L*. *murinus* (C, n = 15) and *E*. *coli* (D, filled bars, n = 20) or *Enterococcus faecalis* (D, open bars, n = 11) at 25% or more of total bacterial populations. Score distributions in C, but not D, are significantly different from those in B (p values of <0.001, 0.17, and 0.16 respectively, χ^2^ test).

The overall occurrence of NEC in the formula feeding-hypoxia group was 60% (28 out of 47 animals), which is in line with previous findings. Average logarithms of *Lactobacillus* loads did not significantly differ between healthy (NEC score 0–1) and sick (NEC score 2–4) animals (Fig 2A). However, prevalence (25% or more of total bacterial population) of *L*. *murinus* HF12, but not of *E*. *coli* or *Enterococcus faecalis*, the two other frequently found species of bacteria, positively correlated with lower NEC scores (Fig 2B–2D). These data suggest that prevalence of *L*. *murinus* HF12 in populations of intestinal bacteria, rather than high absolute load of these bacteria in the intestine, was associated with low scores of NEC.

### The isolated *Lactobacillus* strains are capable of colonizing the neonatal intestine

**Fig 3:**
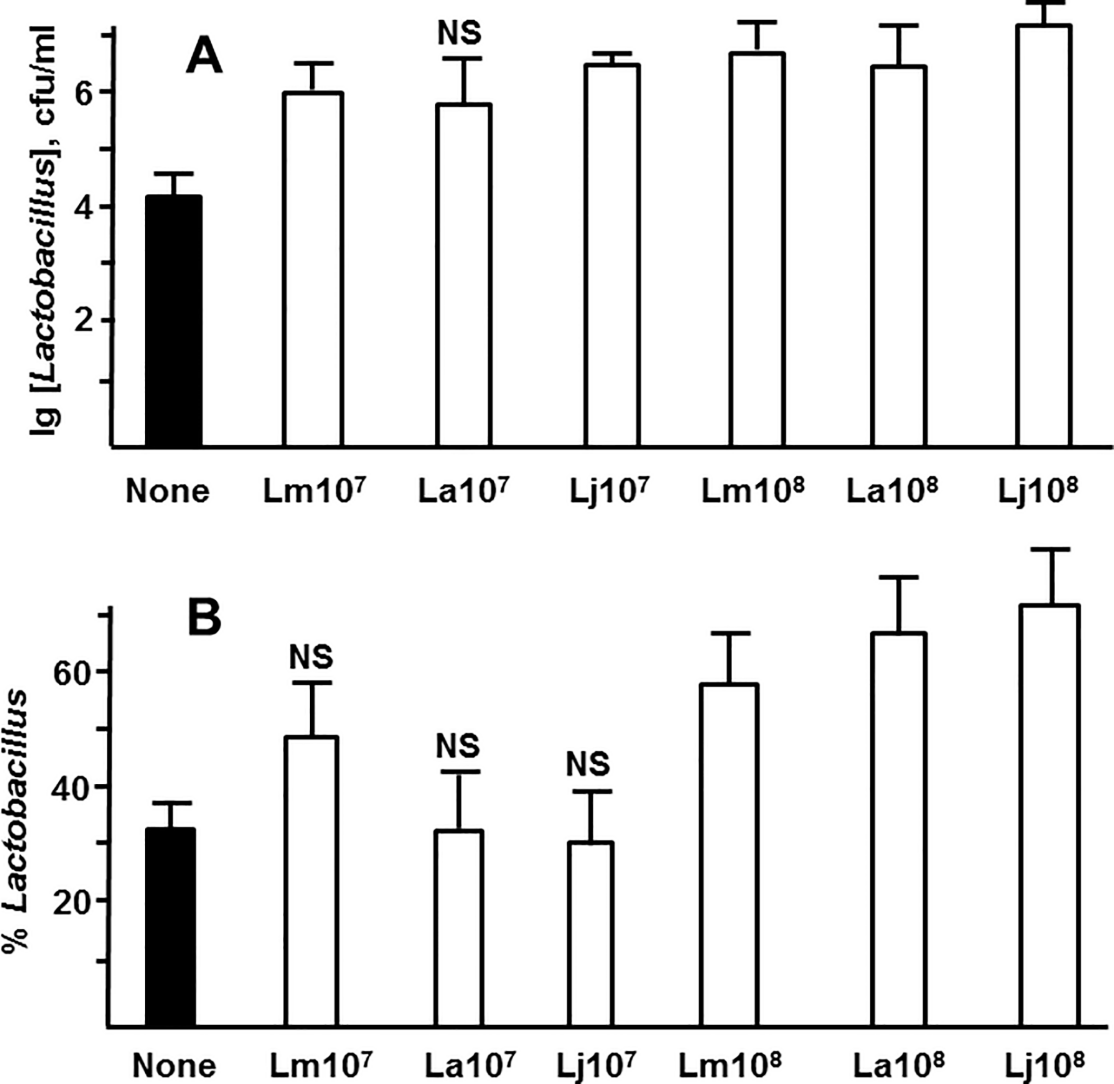
Lactobacillus loads and percentages following inoculation. The indicated lactobacilli were inoculated with first feeding at the indicated dose in cfu per animal. On day 4, total loads and percentages of the inoculated strains in bacterial populations of the intestine were determined. A, average decimal logarithms of *Lactobacillus* concentrations. B, average percentages of Lactobacillus strains in bacterial populations. Lm, La, Lj, *L*. *murinus*, *L*. *acidophilus*, *L*. *johnsonii*, respectively. Filled bars, no inoculation control. NS, non-significant difference from control; other significantly different (unpaired *t* test, p<0.05, n≥9 in each group).

Since early bacterial populations of the gut are inherently transient, we define “colonization” in this study as presence of certain bacteria in substantial numbers and percentages on day 4 of life. In order to determine whether the isolated *Lactobacillus* strains are capable of colonizing the intestine, we grew *L*. *murinus* HF12, *L*. *acidophilus* HF20, and *L*. *johnsonii* HF57 in pure cultures and introduced them to newborn rats with the first feeding at 10^6^–10^9^ cfu, followed by the NEC-inducing regimen of formula feeding-hypoxia for the next 4 days. Inoculation with first feeding was used to mimic early exposure of formula fed neonates to maternal lactobacilli. On day 4, intestinal bacteria were analyzed by plating on blood agar and MRS agar. The recovered lactobacilli were identified using microscopy, colony morphology, and 16S RNA sequencing. 10^6^ cfu doses did not result in significantly higher colonization with the lactobacilli (data not shown). Compared to the control group that was not artificially inoculated, the inoculated groups (except *L*. *acidophilus* at 10^7^ cfu) had significantly greater absolute levels of lactobacilli (Fig 3A, S2 Data File). Inoculation with 10^8^, but not with 10^7^ cfu also significantly increased average percentage of lactobacilli in bacterial populations (Fig 3B). Inoculation with 10^9^ cfu *L*. *murinus* HF12 resulted in inconsistent colonization: some animals had very high levels of *L*. *murinus* on day 4, whereas others had low levels (S2 Data File). According to these results, early artificial introduction of *L*. *murinus* HF12, *L*. *acidophilus* HF20, or *L*. *johnsonii* HF57 at 10^8^ cfu resulted in sizable colonization with these strains on day 4, but in most cases did not prevent naturally occurring colonization with other bacteria.

### *L*. *murinus* HF12 protects from spontaneous NEC

Having established that the three *Lactobacillus* strains are capable of colonizing the intestine of neonatal rats, we evaluated the effects of artificially introduced lactobacilli on NEC. Terminal ileum samples collected on day 4 of the formula feeding-hypoxia regimen were scored for NEC. Introduction of *L*. *murinus*, but not *L*. *johnsonii* or *L*. *acidophilus*, significantly decreased NEC scores compared to control groups at both 10^7^ and 10^8^ cfu (Table 1, S2 Data File). Thus, despite the fact that *L*. *murinus*, *L*. *acidophilus*, and *L*. *johnsonii* were all capable of colonizing the neonatal intestine, only *L*. *murinus* significantly protected the neonates from NEC.

**Table 1:** Effects of inoculation with lactobacilli on spontaneous NEC scores.

Inoculum	Dose, cfu	NEC score 0	NEC score 1	NEC score 2	NEC score 3	NEC score 4	p^1^	N
None		11	9	21	5	1		47
*L*. *murinus*	10^7^	9	6	1	0	0	0.009	16
*L*. *murinus*	10^8^	9	6	1	0	0	0.009	16
*L*. *acidophilus*	10^7^	1	1	5	1	0	0.52	9
*L*. *acidophilus*	10^8^	2	4	6	1	0	0.88	13
*L*. *johnsonii*	10^7^	1	2	6	0	0	0.70	10
*L*. *johnsonii*	10^8^	2	2	5	1	0	0.77	10

^1^Compared to the no inoculum cohort, χ^2^ test.

### *L*. *murinus* HF12 protects from NEC upon challenge with *Cronobacter muytjensii*

*C*. *muytjensii* 51329 (previously classified as *C*. *sakazakii*) is an opportunistic pathogen associated with clinical NEC and neonatal meningitis. It has also been shown to promote NEC in neonatal rats [18]. As expected, *C*. *muytjensii* 51329 increased NEC pathology when introduced to neonatal rats at a single 10^6^ cfu dose with first feeding (p = 0.012, n = 28, χ^2^ test). To elucidate whether the isolated *Lactobacillus* strains can counter the effect of a NEC pathogen, we introduced them with first feeding prior to challenge with *C*. *muytjensii* at the second feeding, and examined changes in NEC pathology.

Introduction of *L*. *murinus* HF12 at 10^8^ cfu/animal prior to treatment with *C*. *muytjensii* significantly decreased NEC scores compared to treatment with *C*. *muytjensii* alone (Table 2). At the same dose, neither *L*. *acidophilus* HF20, nor *L*. *johnsonii* HF57 significantly decreased NEC pathology upon challenge with *C*. *muytjensii* (Table 2). *L*. *murinus* inactivated by heating at 90°C for 10 min failed to significantly protect against NEC (Table 2). Thus, only live *L*. *murinus* HF12 protected from NEC upon challenge with a known opportunistic pathogen.

**Table 2:** Effects of inoculation with lactobacilli on NEC scores upon challenge with 10^6^ cfu *C*. *muytjensii*.

Inoculum	Dose, cfu	NEC score 0	NEC score 1	NEC score 2	NEC score 3	NEC score 4	p^1^	N
None		1	3	18	3	3		28
*L*. *murinus*	10^8^	2	9	3	2	0	<0.001	16
*L*. *murinus*	10^8^	3	4	8	1	0	0.77	16^2^
*L*. *acidophilus*	10^8^	1	3	6	1	0	0.27	11
*L*. *johnsonii*	10^8^	2	2	9	0	1	0.74	14

^1^Compared to cohort challenged with *C*. *muytjensii* without inoculation with lactobacilli, χ^2^ test.

^2^Heat-inactivated *L*. *murinus*.

### *L*. *murinus* reduces molecular markers and intestinal barrier damage of NEC

**Fig 4:**
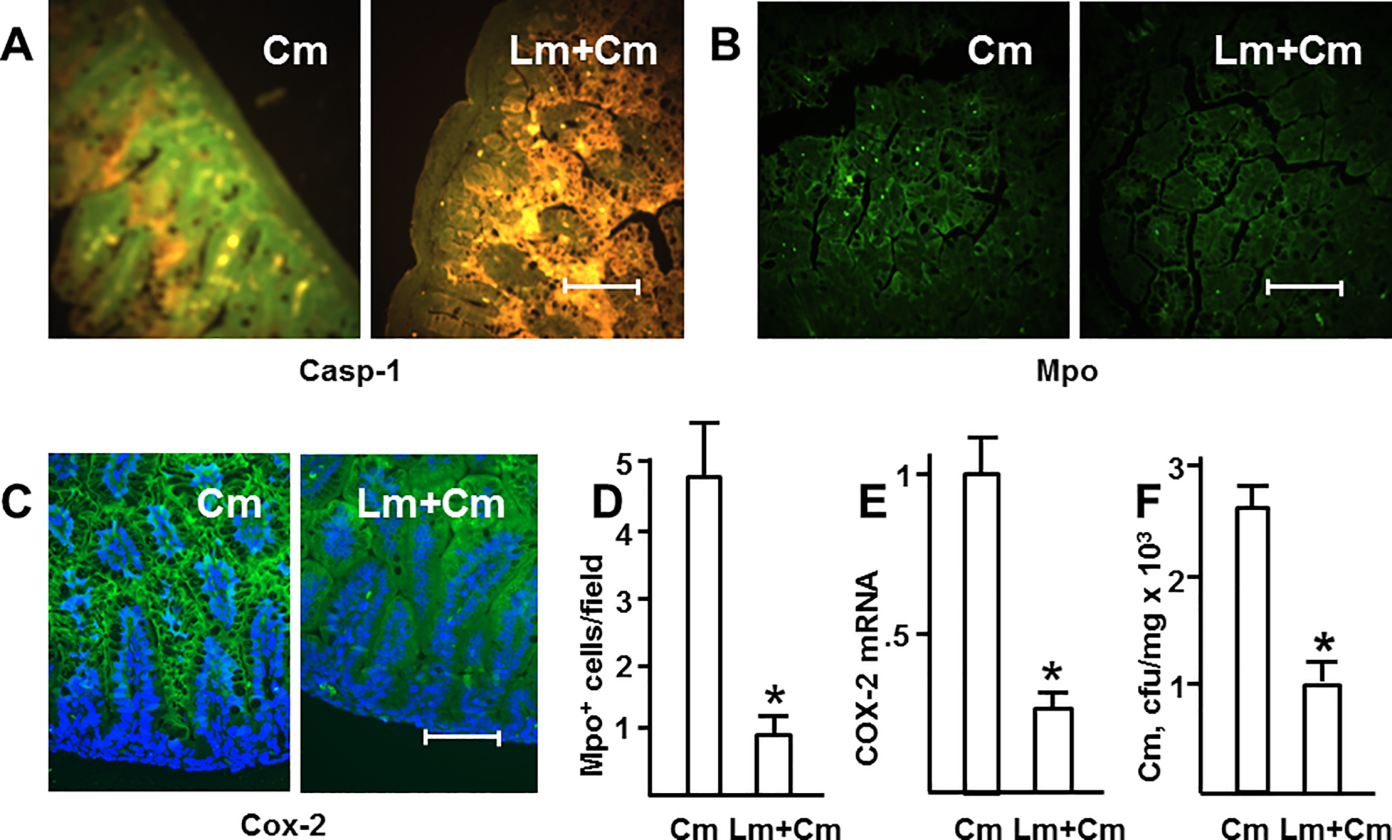
*L*. *murinus* reduces molecular markers and intestinal barrier damage of NEC. Localization of active caspase-1 (green, A), myeloperoxidase-positive cells (green, B), and Cox-2 (green, C) in small intestine sections of 4 day old animals inoculated with *C*. *muytjensii* (Cm) at 10^6^ cfu at the second feeding, without or with pre-inoculation with *L*. *murinus* HF12 at 10^8^ cfu at the first feeding. Yellow/orange/brown in (A) is non-specific autofluorescence; blue in (C) are DAPI-stained nuclei. Each image is representative of at least 3 animals. Bar = 100 μm. D, average numbers of Mpo-positive cells in random 200x200 μm squares (n = 16 in each group). E, relative levels of mucosal COX-2 mRNA (n = 4 in each group). F, loads of *C*. *muytjensii* per mg wet weight aseptically excised spleen tissue (n = 10 in each group). Average intestinal loads of *C*. *muytjensii* in the two groups were not significantly different (8.2±1.2 vs. 7.6±2.0 x 10^5^ cfu/ml, p = 0.8). *, significant differences (p<0.01, unpaired *t* test).

NEC is associated with elevated expression of several inflammatory markers and derangement of the epithelial barrier. To elucidate whether *L*. *murinus* attenuates these changes, we examined activation of caspase-1, recruitment of myeloperoxidase (Mpo)-positive cells, expression of cyclooxygenase-2 (COX-2), and translocation of luminal bacteria to spleen. Caspase-1 facilitates processing and secretion of IL-1β, therefore active caspase-1 indicates IL-1β release. Mpo is a marker of neutrophils, whose recruitment to the intestine is characteristic of NEC. Cox-2, a rate-limiting enzyme in the production of inflammatory prostanoids, is also one of the key markers of intestinal inflammation. We used the standard formula feeding–hypoxia model enhanced by challenging with 10^6^ cfu *C*. *muytjensii* during second feeding, without or with pre-inoculation with 10^8^ cfu *L*. *murinus* HF12 during first feeding. *L*. *murinus* reduced or completely abrogated activation of intestinal caspase-1 (Fig 4A), attenuated recruitment of Mpo-positive cells to the intestine (Fig 4B and 4D), and reduced epithelial COX-2 immunofluorescence (Fig 4C) as well as tissue levels of COX-2 mRNA (Fig 4D). *L*. *murinus* significantly reduced translocation of *C*. *muytjensii* from intestinal lumen to spleen (Fig 4F). Thus, *L murinus* reduced not only microscopic pathology, but also molecular and physiologic manifestations of NEC.

## Discussion

We have isolated three strains of lactobacilli belonging to three different species from the intestines of 4-day old rats. Lactobacilli were found in the majority of animals, and were often the predominant group in the populations of intestinal bacteria. *L*. *murinus* was the most frequently isolated *Lactobacillus*, which is in agreement with the reported ubiquitous prevalence of this species in rodents [24]. Introduction of the pure cultures of *L*. *murinus* HF12, *L*. *acidophilus* HF20, or *L*. *johnsonii* HF 57 to rat neonates resulted in successful colonization, demonstrating that each of these strains is capable of colonizing the neonatal intestine. The ability of *L*. *murinus* to colonize the stomach and esophagus of germ-free mice has been previously described [25]. Although the three *Lactobacillus* strains were successful as early colonizers in our experiments, neither of them, even when introduced in relatively high numbers, prevented spontaneous colonization with other bacteria. Thus, we have identified three naturally occurring strains of lactobacilli that are capable of colonizing the GI tract upon introduction to newborn rats. We have also demonstrated the possibility of skewing bacterial populations towards lactobacilli, but not mono-association, by early artificial introduction of these bacteria to the neonates.

Although *L*. *murinus* HF12, *L*. *acidophilus* HF20, and *L*. *johnsonii* HF57 were all successful first colonizers, only *L*. *murinus* significantly protected neonatal rats from NEC, according to three lines of evidence. First, high proportions of *L*. *murinus* HF12 in bacterial populations were associated with low NEC scores in the original cohort of 51 animals subjected to the formula feeding–hypoxia regimen. Second, introduction of *L*. *murinus* HF12 during the first feeding significantly reduced NEC scores in the formula feeding–hypoxia model. Third, introduction of *L*. *murinus* HF12 protected from NEC upon challenge with the known NEC pathogen, *C*. *muytjensii*. Our study satisfies all the four Koch’s requirements (postulates) to establish connection between specific microbe and disease. First, high proportion of *L*. *murinus* HF12 was found in animals with low scores of NEC. Second, this strain was isolated in pure culture. Third, introduction of this strain to the neonates significantly protected from NEC. Fourth, animals inoculated with *L*. *murinus* HF12 were found to harbor this strain 4 days after the introduction. The lack of protection by the strains of *L*. *acidophilus* and *L*. *johnsonii* indicates that different members of the *Lactobacillus* genus may differ in their ability to prevent NEC. Since we have identified only one strain of *L*. *murinus* in this study, it remains unclear whether other strains of this species possess similar protective properties.

Our study suggests a rational approach to manipulation of early intestinal microbiota for the purpose of preventing NEC. Early introduction of beneficial colonizing bacteria to pre-term neonates may skew the emerging bacterial populations towards non-pathogenic microbiota and thus protect the intestine. Although mono-colonization with the artificially introduced lactobacilli is unlikely, our data show that skewing bacterial populations towards lactobacilli is a feasible task. Strains of lactobacilli (and/or other commensal bacteria) suitable for such intervention should satisfy the following three characteristics: 1) common occurrence in healthy neonates; 2) competitiveness as first colonizers of the intestine; and 3) ability to protect from NEC. Our results may be not directly applicable to clinical NEC, which is an obvious limitation of this study. The timeline of human NEC and typical first colonizers are vastly different from those of rat NEC [26]. However, the principle of protection from NEC by artificial introduction of beneficial colonizing bacteria, which we validate here, is clinically relevant. Identifying clinically relevant beneficial first colonizers is an important step in developing this approach.

## Supporting information

S1 Data File Bacterial populations in 4 day old rats. (XLSX)

S2 Data File Bacterial populations following introduction of lactobacilli. (XLSX)

S1 Fig Representative NEC pathology images. Numbers are NEC scores: 0, intact epithelium; 1, epithelial sloughing; 2, destruction of tips of the villi; 3, destruction of whole villi; 4, obliteration of the epithelium. The images are of hematoxylin-eosin-stained terminal ileum sections of newborn rats subjected to 4 days of formula feeding–hypoxia. Animals were inoculated with 10^8^ cfu *L*. *murinus* (Lm) at first feeding and/or 10^6^ cfu *C*. *muytjensii* (Cm) at second feeding as indicated. Bar = 100 μm.
(TIF)
