# Supplementary figures and images for: Lactobacillus murinus HF12 colonizes neonatal gut and protects rats from necrotizing enterocolitis
Source: PLoS One. 2018 Jun 22;13(6):e0196710. doi: 10.1371/journal.pone.0196710 (PMC6014650; doi:10.1371/journal.pone.0196710)

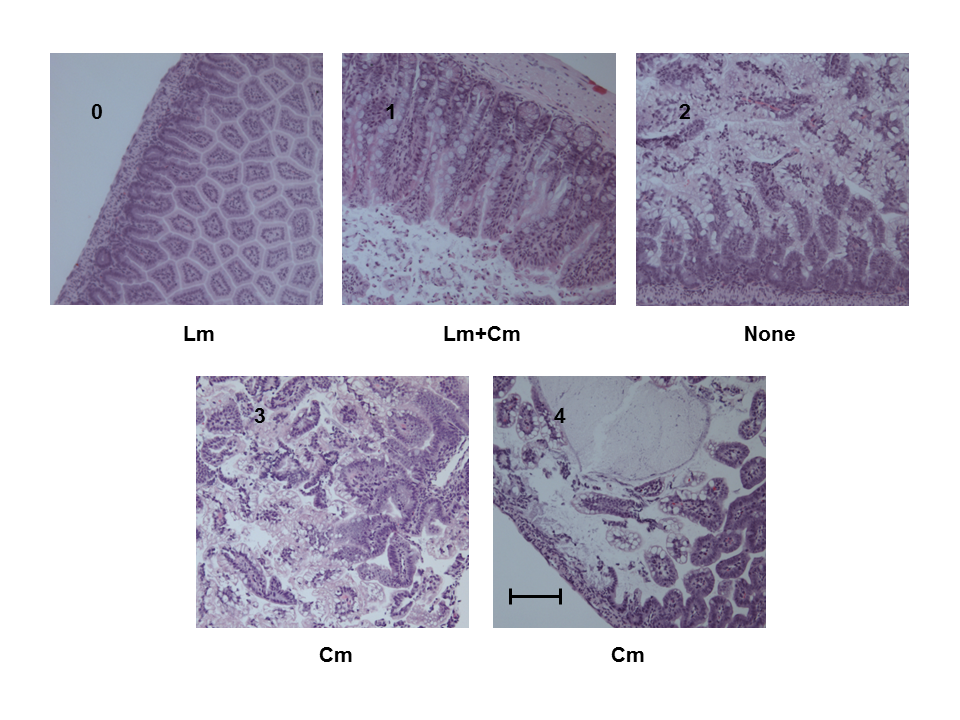

Supplement: S1 Fig — Numbers are NEC scores: 0, intact epithelium; 1, epithelial sloughing; 2, destruction of tips of the villi; 3, destruction of whole villi; 4, obliteration of the epithelium. The images are of hematoxylin-eosin-stained terminal ileum sections of newborn rats subjected to 4 days of formula feeding–hypoxia. Animals were inoculated with 108 cfu L. murinus (Lm) at first feeding and/or 106 cfu C. muytjensii (Cm) at second feeding as indicated. Bar = 100 μm. (TIF) [file pone.0196710.s003.tif]
